# Retinal phototoxicity in a novel murine model of intraocular lens implantation

**Published:** 2009-12-12

**Authors:** Toshihide Kurihara, Masahiro Omoto, Kousuke Noda, Mari Ebinuma, Shunsuke Kubota, Haruna Koizumi, Satoru Yoshida, Yoko Ozawa, Shigeto Shimmura, Susumu Ishida, Kazuo Tsubota

**Affiliations:** 1 Laboratory of Retinal Cell Biology, Keio University School of Medicine, Tokyo, Japan; 2 Department of Ophthalmology, Keio University School of Medicine, Tokyo, Japan; 3 Laboratory of Corneal Cell Biology, Keio University School of Medicine, Tokyo, Japan; 4 Department of Ophthalmology, Hokkaido University Graduate School of Medicine, Hokkaido, Japan

## Abstract

**Purpose:**

To establish a novel murine intraocular lens (IOL) implantation model to study the protective effects of colored-IOLs against retinal phototoxicity.

**Methods:**

Two-millimeter diameter IOL buttons were created from IOLs for clinical use. Extra-capsular crystalline lens extraction and IOL implantation were performed in BALB/c mice using a technique similar to human cataract surgery. For light exposure experiments, mice were exposed to 5,000 LUX of white light for 24 h on the day after surgery. To investigate the protective effects of yellow IOL against light exposure, ERG measurements were conducted in vivo, followed by TdT-mediated dUTP Nick-End Labeling (TUNEL) and outer nuclear layer (ONL) thickness measurement of retinal tissue in yellow or clear IOL-implanted mice and control mice without surgery.

**Results:**

IOLs were successfully implanted in all animals, and IOL buttons without haptics were well stabilized in the capsular bag. Murine eyes developed posterior capsule opacification (PCO) after IOL implantation by postoperative day 5 at the latest. In contrast to the clear IOL-implanted animals stimulated by light exposure, the yellow IOL-implanted animals had significantly reduced numbers of TUNEL-positive cells and retained thickness of the ONL. The ERG showed that yellow IOL implantation prevents a decrease of amplitude in both the a-wave and b-wave compared with clear IOL implantation.

**Conclusions:**

We established a new animal model of IOL implantation and demonstrated the protective effects of colored-IOL against retinal phototoxicity after cataract surgery.

## Introduction

Improvements in cataract surgery lead to better postoperative vision with lower complication rates; however, visual outcomes may still be compromised by certain postoperative changes, such as posterior capsule opacification (PCO) and cystoid macular edema [1,2]. In addition, the change in light transmission range between pre- and post-cataract surgery is thought to be associated with another postoperative complication: development of age-related macular degeneration (AMD) [3]. AMD is the leading cause of severe vision loss in people over age 50 in developed countries [4]. Several lines of evidence indicate that retinal phototoxicity, which induces reactive oxygen species and results in tissue damage, is involved in the development of AMD after cataract surgery [5]. Under normal conditions, the sensory retina is protected from ultraviolet light (UV, 100–400 nm) by the cornea and crystalline lens [6]. Aging can also cause the crystalline lens to filter higher frequency visible light (400–500 nm), i.e., violet or blue [7]. Therefore, crystalline lens extraction during cataract surgery enables UV and short wavelength visible light, which are implicated in phototoxicity, to reach the retina.

The Beaver Dam Eye Study showed that cataract surgery was associated with a fourfold increase in the risk of neovascular AMD and a threefold increase in geographic atrophy 10 years later [8,9]. Similar findings were reported in the Blue Mountains Eye Study [10,11]. In accordance with the clinical data, it was shown in vitro that blue light (441 nm), which is in the short wavelength of visible light, is the most harmful to photoreceptors and the retinal pigment epithelium (RPE) [12]. Thus, it is likely that retinal phototoxicity is one of the pivotal causes of AMD.

Elucidating the mechanisms underlying the postoperative threats to vision relevant to cataract surgery has been of great interest, and several experimental animal models have been established using in vivo and in vitro techniques for that purpose [13,14]. In the past, rabbit and rat models in which the animals wore an external, colored filter absorbing blue light were used to evaluate the protective effect of yellow-tinted intraocular lenses (IOL) against white light [15,16]. Since the rat crystalline lens, for example, stably transmits a significant percentage of light with wavelengths ranging from 350 nm to the end of the visible spectrum [16], short wavelength light can transmit in the presence of the crystalline lens. Therefore, in the case of rat eyes, it may not be necessary to remove the crystalline lens to conduct the phototoxicity study [16]. However, since the rat crystalline lens still absorbs part of the blue light [16], this led us to the idea that an animal model with lens extraction and IOL insertion may be a more realistic model for the investigation of retinal phototoxicity after cataract surgery. Furthermore, IOL implantation would make possible the evaluation of the protective effect of IOLs with different spectral transmissions against light-induced retinal degeneration.

In the current study, we establish a novel murine IOL implantation model and study the impact of a colored-IOL on retinal phototoxicity compared with a clear IOL. Our murine model, involving both crystalline lens removal and IOL implantation, mimics more closely the conditions after human cataract surgery.

## Methods

### Animals

A total of 83 BALB/c male mice (6 weeks old; n=17 for control and 66 for IOL implantation; Clea, Tokyo, Japan) were used in this study. Animals were housed in plastic cages in a climate-controlled animal facility and kept under dim cyclic light (5 LUX, 12 h on/off) in our institution, except where otherwise indicated. All animal experiments were conducted in accordance with the Association for Research in Vision and Ophthalmology (ARVO) Statement for the Use of Animals in Ophthalmic and Vision Research.

### IOLs

A conventional clear IOL (SA60AT) or blue light- and UV-absorbing yellow IOL (SN60AT) was used for the following experiments [17]. The IOLs were kindly given by Alcon Laboratories, Inc. (Fort Worth, TX). While the clear IOL transmits more than 90% of the 400 nm and longer wavelengths, it blocks ultraviolet (UV) light [18]. In contrast, the yellow IOL also absorbs wavelengths corresponding to blue light (400–500 nm), similar to the spectral transmittance of the adult human crystalline lens [18]. Compared with the clear IOL, the yellow IOL reduces 71% of the transmittance at 400 nm [18].

### Extra-capsular crystalline lens extraction and IOL implantation

**Figure 1:**
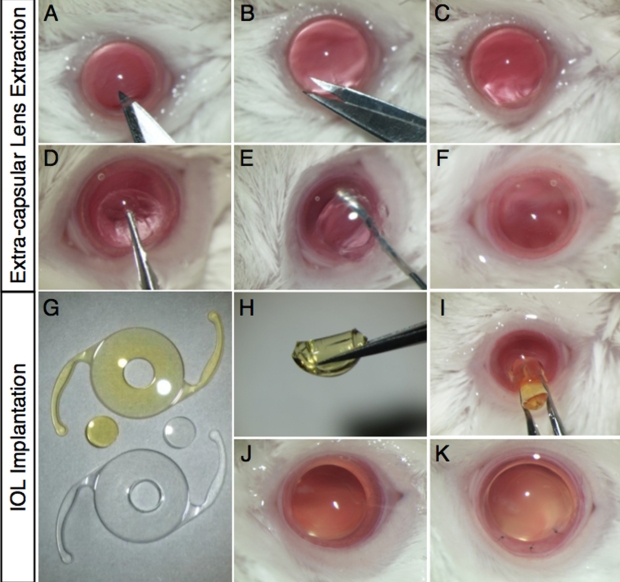
Extra-capsular crystalline lens extraction and IOL implantation in murine eyes. **A**-**C**: Corneal incision with a surgical knife and Vannas scissors. **D**: Continuous curvilinear capsulorrhexis with forceps. **E**: Lens extraction with hydrodissection. **F**: Inflation of the capsular bag with a viscoelastic substance. **G**: Fabricated IOL buttons (2 mm diameter). **H**, **I**: IOL insertion. **J**, **K**: Removal of the viscoelastic substance and closure of the corneal wound with interrupted 11–0 nylon sutures.

Mice were anesthetized with an intramuscular injection of a mixture of 80 mg/kg Ketamine and 16 mg/kg Xylazine before surgical procedures. Pupils were dilated using 0.5% Tropicamide and 0.5% Phenylephrine Hydrochloride (Mydrin-P; Santen Pharmaceutical, Osaka, Japan). The operation was conducted under a surgical microscope (Leica MZ16, Leica Microsystems, Wetzlar, Germany) with a light source (Leica CLS 150 XD, Leica), and the surgical time was approximately 15 min per eye. Paracentesis was performed with a 22.5° knife (MST22; MANI, Tochigi, Japan; Figure 1A), and 1% Sodium Hyaluronate (OPEGAN Hi; Santen Pharmaceutical, Osaka, Japan) was injected into the anterior chamber. The corneal incision was extended to approximately 90° using Vannas scissors (Geuder, Heidelberg, Germany; Figure 1B,C), and anterior curvilinear continuous capsulorrhexis was made with Jewelers Forceps (Katena, Denville, NJ; Figure 1D). Thereafter, hydrodissection was performed with a 30-gauge cannula to deliver the lens en bloc (Figure 1E). The lens cortex was aspirated through the cannula (Figure 1F).

The central portion of the IOL optic was trephined with a 2 mm diameter biopsy punch (Kai, Gifu, Japan; Figure 1G). The IOL button was folded by forceps (Figure 1H) and inserted into the capsular bag through the corneal incision (Figure 1I). After IOL insertion, residual viscoelastic material was replaced with phosphate buffered saline (PBS; Figure 1J). To close the corneal wound, 3 to 5 interrupted 11–0 nylon sutures (0550s; MANI) were placed (Figure 1K). To prevent postoperative inflammation and infection, we administered topical antibiotics (levofloxacin) and corticosteroids (betamethasone) at the end of the surgery. The animals with endophthalmitis or IOL implantation failure were excluded from the study. All of the surgeries were performed by the same investigator (M.O.) and involved one eye per animal – the right eye.

In this new model, to clarify the time course of PCO development, one of the most frequent postoperative complications of cataract surgery, paraffin sections of IOL-implanted eyes were prepared using standard techniques. Hematoxylin and eosin (H&E) staining was also performed. Briefly, at 1 and 5 days after IOL implantation, eyes were enucleated from the animals under deep anesthesia and fixed in 4% paraformaldehyde (PFA). After fixation, tissues were processed and embedded in paraffin. Five-µm sections from the middle portion of the eye were prepared and stained with H&E.

### Light exposure

For light exposure experiments, mice were exposed to 5,000 LUX of white light for 24 h in a dedicated exposure box, having stainless mirrors at the lateral sides and floors (Tinker-N, Kyoto, Japan). The box contained a white fluorescent lamp (FHD100ECW, Panasonic, Osaka, Japan) and an air conditioner unit to maintain the temperature inside at 23 °C. Prior to the light exposure, mice were dark adapted for 12 h. The pupils were then dilated with a mixed solution of 0.5% Tropicamide and 0.5% Phenylephrine just before the light exposure.

### TdT-mediated dUTP nick-end labeling (TUNEL)

Mice were anesthetized with pentobarbital sodium (70 mg/kg BW) and perfused with 10 ml of PBS 48 h after the start of light exposure. Subsequently, eyes were enucleated and fixed in 4% PFA overnight at 4 °C. After fixation, tissues were processed and embedded in an OCT compound (Tissue-Tek, Sakura, CA) for cryosections. Six 10 µm cryosections from the optic nerve were prepared, and TUNEL was performed using the ApopTag Red apoptosis detection kit (Chemicon, CA) according to the manufacturer's protocol. Nuclei were stained with 10 µg/ml Hoechst bisbenzimide 33258 (Sigma-Aldrich, MO). Fluorescence images were obtained using Axio Imager (Carl Zeiss, Oberkochen, Germany), and TUNEL-positive cells were counted in the outer nuclear layer (ONL) of the sections, including the optic nerve head.

### Measurement of outer nuclear layer thickness

Mice were anesthetized with pentobarbital sodium (70 mg/kg BW), perfused with 10 ml of PBS 48 h after the start of light exposure, and the eyes were then enucleated. Paraffin-embedded retinal sections (3 µm) were prepared and stained with H&E. ONL thickness was measured at each 0.2 mm point from the optic nerve head to the most peripheral area using ImageJ software (National Institutes of Health, Bethesda, MD), as described previously [16].

### Electroretinography

Electroretinography (ERG) analysis was performed as previously described, 48 h after the start of light exposure [19,20]. Responses were differentially amplified and filtered through a digital bandpass filter ranging from 0.313 to 1,000 Hz to yield a- and b-waves. Light pulses of 800 cd·s/m^2^ and 4 ms duration were delivered via a commercial Ganzfeld stimulator (Ganzfeld System SG-2002; LKC Technologies, Inc.). The amplitude of the a-wave was measured from the baseline to the trough of the a-wave, and the amplitude of the b-wave was determined from the trough of the a-wave to the peak of the b-wave. The implicit time of the a- and b-waves was measured from the onset of stimuli to the peak of each wave.

### Statistical analysis

All results are expressed as mean±SD with n-numbers as indicated. The student’s *t*-test was used for statistical comparison between the groups. Differences between the means were considered statistically significant when the probability values were <0.05.

## Results

### Establishment of an experimental IOL implantation model in mice

To mimic the conditions of human cataract surgery and its effects on the retina, we developed a murine model of IOL implantation. The surgical procedures for IOL implantation in mice performed in this study were mostly identical to those of human cataract surgery (Figure 1). Two-mm diameter IOL buttons were created from the IOL for clinical use, and the IOL button without haptics was well stabilized in the capsular bag. Throughout the present study, IOLs were successfully implanted in all the animals.

**Figure 2:**
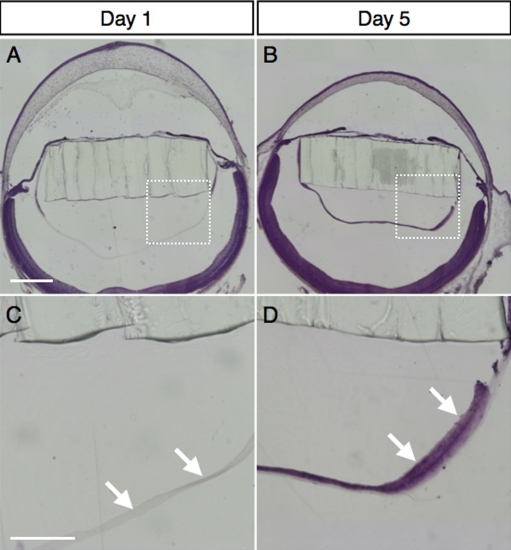
Development of posterior capsular opacity in murine IOL-implanted eyes. Representative micrographs of murine IOL-implanted eyes, stained with hematoxylin and eosin on post-operative day 1 (**A**) or day 5 (**B**) and high-magnification images of the posterior lens capsule (**C**, **D**; square area marked in **A** and **B**, respectively). The arrows depict lens epithelial cells migrating underneath the posterior capsule on post-operative day 5 (**D**), whereas no cells were seen on post-operative day 1 (**C**). The bar shown in **A** represents 500 µm and applies for (**B**) as well. The bar shown in **C** represents 200 µm and applies for (**D**) as well.

Morphological study revealed that the murine eyes developed PCO after IOL implantation (Figure 2). Whereas no cells existed underneath the posterior capsule on postoperative day 1 (Figure 2A,C), lens epithelial cell migration was observed at postoperative day 5 (Figure 2B,D), indicating that PCO developed a few days after the surgery in this model. No difference was seen in lens epithelial cell migration between clear IOL and yellow IOL implantation (data not shown).

### Effects of IOL implantation on the retinal tissues

To determine whether IOL implantation itself impairs the ocular tissues, particularly the posterior segment of the eyes, we examined structural and functional changes in the retinal tissues after IOL implantation.

**Figure 3:**
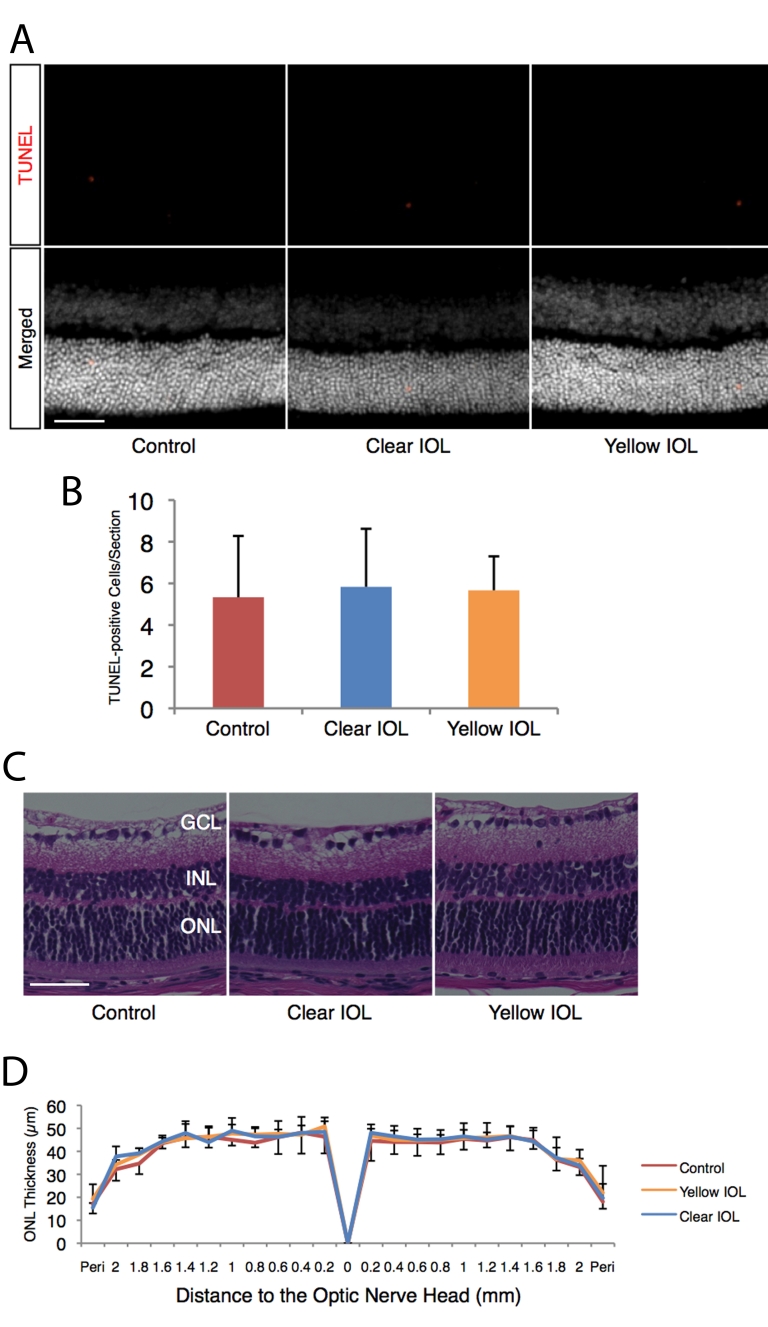
Morphological assessment of sensory retina in murine IOL-implanted eyes and control eyes without surgery. Apoptotic photoreceptors and ONL thickness after IOL implantation were assessed under dim light conditions (5 LUX, 12 h on/off). **A**: (Upper) Representative images of TUNEL staining for retinal sections 1 mm superior to the optic nerve head at postoperative day 3. (Lower) Merged images. Nuclei were counterstained with Hoechst 33258. Bar=50 µm. **B**: Quantification of TUNEL-positive cells in the ONL of each section, including the optic nerve head. Values are mean ±SD (n=6 in each group). **C**: Representative images of hematoxylin and eosin staining for retinal sections 1 mm superior to the optic nerve head at postoperative day 7. Bar represents 50 µm. **D**: ONL thickness of control or IOL-implanted eyes. Values are mean ±SD (n=5 to 6 in each group).

First, we studied the apoptotic changes in the retina after IOL implantation. At 3 days after the surgery, the numbers of TUNEL-positive cells in clear IOL-implanted eyes (5.8±2.8 cells/section, n=6) showed no significant difference compared to eyes without IOL implantation (5.3±2.9 cells/section, n=6, p=0.811, Figure 3A,B). Similarly, the numbers of TUNEL-positive cells for the clear IOL-implanted eyes resembled those of the yellow IOL-implanted eyes (5.6±1.6 cells/section, n=6, p=0.908, Figure 3A,B).

In line with TUNEL, at 7 days after the surgery, the ONL thickness showed no difference between clear IOL-implanted eyes (n=5) and eyes without surgery under dim light (n=6, Figure 3C,D). The ONL thickness in clear IOL-implanted eyes showed no significant difference when compared with that of yellow IOL-implanted eyes (n=6, Figure 3C,D).

**Figure 4:**
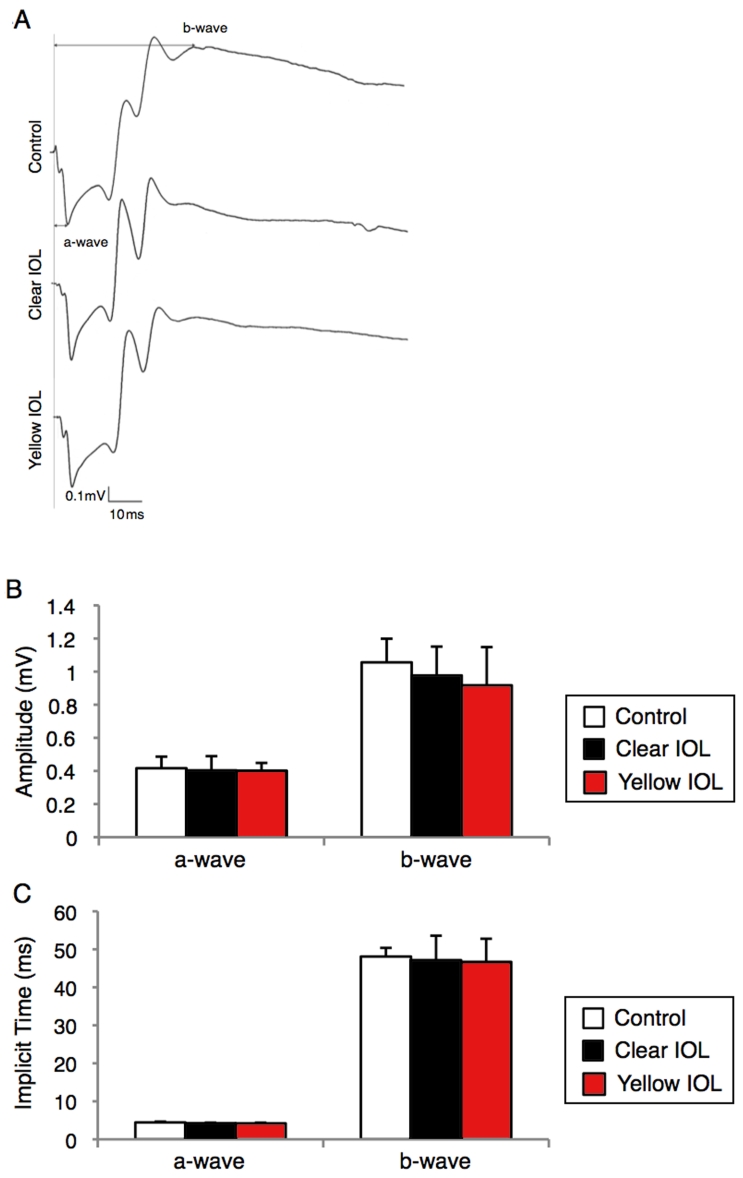
Functional assessment of sensory retina in murine IOL-implanted eyes. ERG was measured under dim (5 LUX, 12 h on/off) light at 7 days after IOL implantation. **A**: Representative wave responses from control or IOL-implanted eyes. **B**, **C**: Quantification of amplitude (**B**) or implicit time (**C**) of a-wave and b-wave. Values are mean ±SD (n=5 in each group).

Furthermore, the ERG amplitude of the a-wave from clear IOL-implanted mice (0.40±0.09 mV, n=5, Figure 4A,B) or yellow IOL-implanted mice (0.40±0.05 mV, n=5, Figure 4A,B) was not different from that of mice without surgery under dim light (0.42±0.07 mV, n=5, p=0.82 for clear IOL, 0.76 for yellow IOL, Figure 4A,B). Similarly, the ERG amplitude of the b-wave from clear IOL-implanted mice (0.98±0.17 mV, n=5, Figure 4A,B) or yellow IOL-implanted mice (0.92±0.23 mV, n=5, Figure 4A,B) was not different from that of mice without surgery under dim light (1.06±0.14 mV, n=5, p=0.41 [compared with clear IOL], p=0.29 [compared with yellow IOL], Figure 4A,B). These data suggest that IOL implantation itself elicits neither morphological nor functional loss in the retinal tissue of murine eyes. The implicit time showed a similar pattern of no differences between the groups regarding amplitude (a-wave: 4.4±0.21 ms for control, 4.2±0.15 ms, p=0.22 [compared with control] for clear IOL, 4.2±0.19 ms, p=0.19 for yellow IOL; b-wave: 48.1±2.3 ms for control, 47.2±6.4 ms, p=0.77 for clear IOL, 46.7±6.1 ms, p=0.51 for yellow IOL, n=5 in each group, Figure 4C).

### Impact of yellow IOL on light-induced retinal degeneration

To investigate the protective effects of yellow IOL against light exposure, we conducted TUNEL, ONL thickness measurement and ERG on retinal tissues of mice that had been IOL-implanted with either the clear or yellow lens and subjected to 5,000 LUX of white light for 24 h. Since H&E staining had demonstrated that the PCO developed 2–3 days after the implantation surgery (Figure 2), we exposed the animals to light stimulation the day after the surgery.

**Figure 5:**
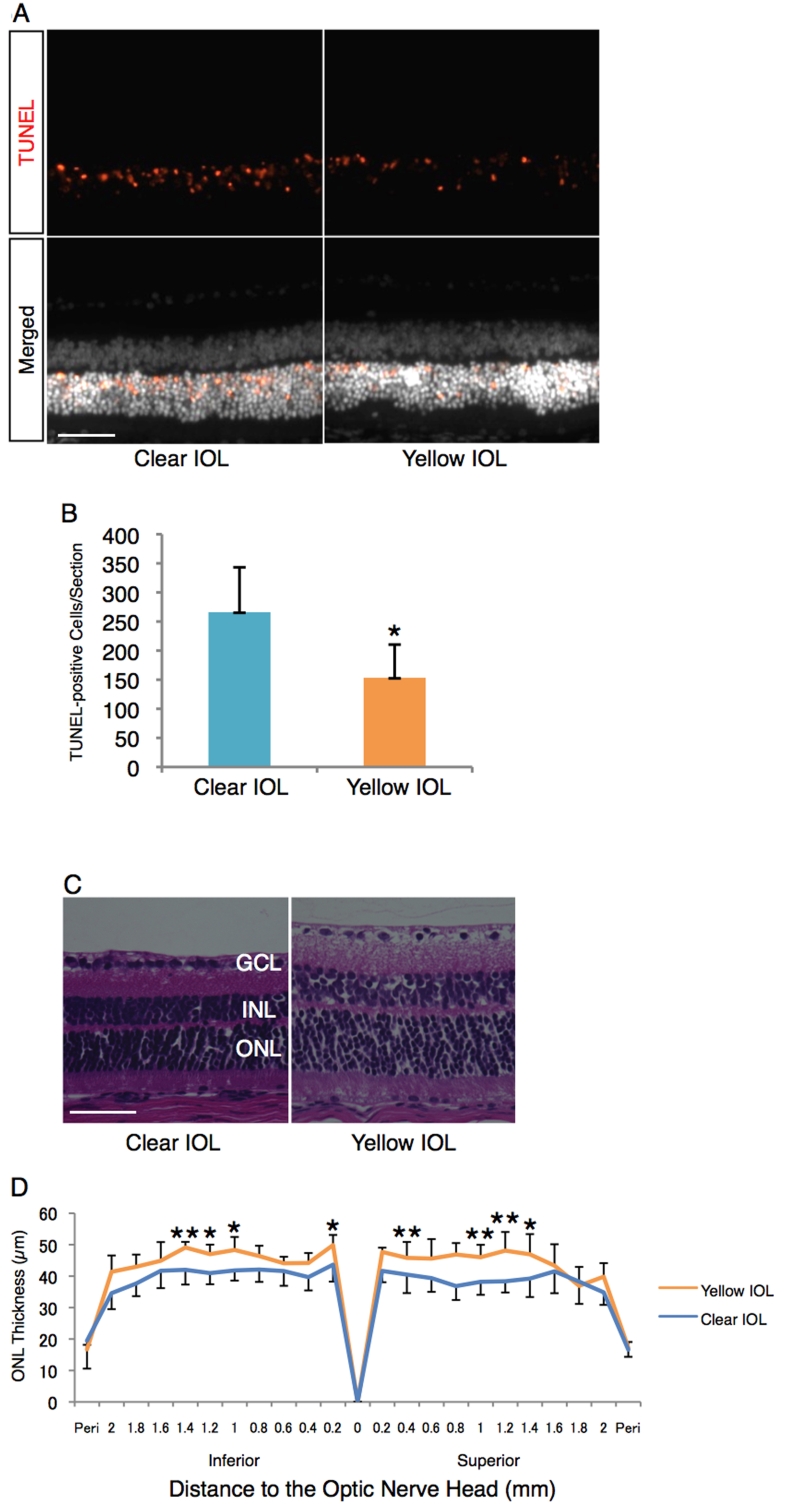
Impact of yellow IOL on the light-induced retinal morphological changes. **A**: (Upper) Representative images of TUNEL staining for retinal sections 1 mm superior to the optic nerve head on postoperative day 3 (2 days after the light exposure [5,000 LUX, 24 h]). (Lower) Merged images. Nuclei were counterstained with Hoechst 33258. Bar represents 50 µm. **B**: Quantification of TUNEL-positive cells in the ONL of each section, including the optic nerve head. Values are mean ±SD (n=6 in each group; *p<0.05). **C**: Representative images of hematoxylin and eosin staining for retinal sections 1 mm superior to the optic nerve head on postoperative day 7 (6 days after the light exposure [5,000 LUX, 24 h]). Bar represents 50 µm. **D**: ONL thickness of IOL-implanted eyes after light exposure. Values are mean ±SD (n=5 to 7; *p<0.05, **p<0.01).

**Figure 6:**
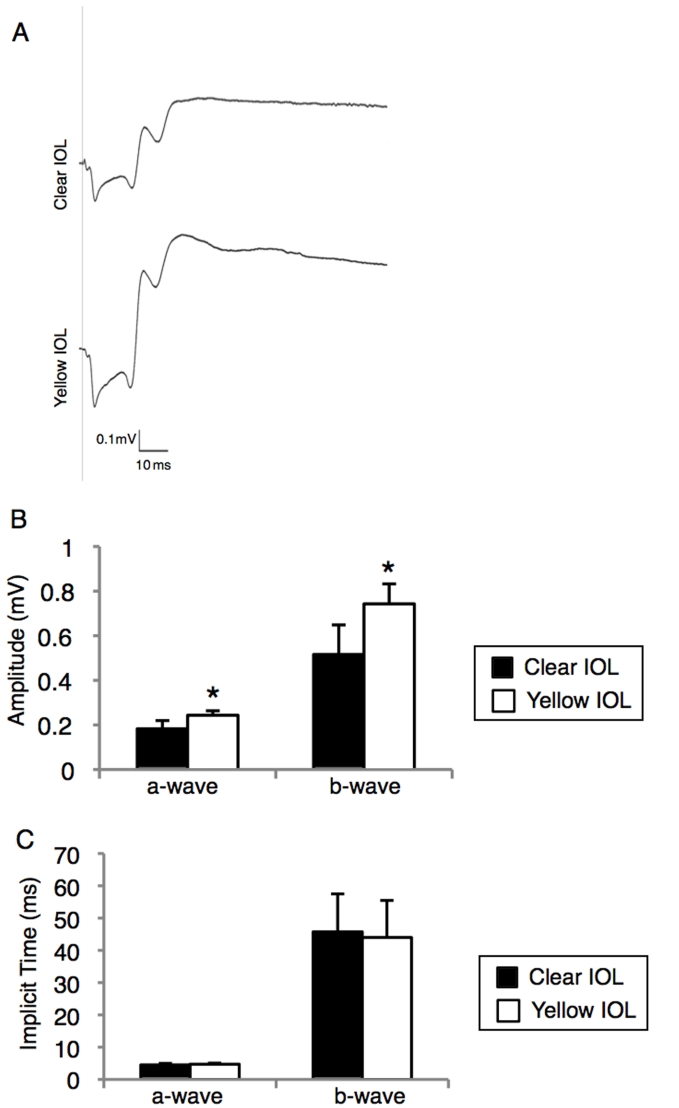
Protective effects of yellow IOL against light-induced retinal functional damage. ERG was measured at postoperative day 7 (6 days after the light exposure [5,000 LUX, 24 h]) in IOL implanted-mice. **A**: Representative wave responses from clear or yellow IOL-implanted eyes. **B**, **C**: Quantification of amplitude (**B**) or implicit time (**C**) of a-waves and b-waves from each group (n=5 in each group, respectively; *p<0.05).

Whereas animals without light exposure showed no or very few TUNEL-positive cells in the retina (Figure 3), there was a dramatically higher number of TUNEL-positive cells in the retina of clear IOL-implanted animals after light exposure (266±77 cells/section, n=6, Figure 5A,B). In contrast to these clear IOL-implanted animals, yellow IOL-implanted animals had significantly reduced numbers of TUNEL-positive cells after light exposure (153±57 cells/section, n=6, p<0.01, Figure 5A,B), yet they also showed higher numbers of cells than animals without exposure. Furthermore, animals with yellow IOL implantation retained the ONL thickness after light exposure compared with clear IOL implantation (n=7 for clear IOL, n=5 for yellow IOL, Figure 5C,D). In line with the apoptotic changes in the retinal tissues, the ERG showed that yellow IOL implantation prevents a decrease of amplitude in the a-wave (0.18±0.04 mV, versus 0.24±0.02 mV, n=5 in each group, p<0.05, Figure 6A,B) and b-wave (0.52±0.13 mV, n=5 versus 0.74±0.09 mV, n=5 in each group, p<0.05, Figure 6A,B) compared with clear IOL implantation after light exposure. The implicit time showed no differences between the groups (p=0.42 in the a-wave, p=0.82 in the b-wave, n=5 in each group, Figure 6C).

## Discussion

In the current study, we established a novel in vivo murine model of IOL implantation. To date, rabbits have generally been used for the in vivo model of IOL implantation [21]. Mice had not yet been used for this purpose, possibly due to the difficulty in performing surgery. To our knowledge, the present study is the first report of an in vivo experiment of IOL implantation in mice, which is virtually identical to human cataract surgery. We found that the surgical procedures had no deleterious effects on the retina and that the phototoxicity data was reproducible. Using this new animal model for IOL implantation, we found that a yellow-tinted IOL protected retinal tissue from phototoxicity.

In this study, we implanted a total of 66 IOL buttons into murine eyes. The surgical procedure to implant the IOLs in murine eyes required some initial practice time to master, but it was then possible to establish reproducible results. Furthermore, we verified that the morphology and function of the sensory retina remained unaffected by the IOL implantation procedure itself. This indicates the stability and utility of this model as an experimental tool for cataract surgery.

The IOL-implanted eyes in our model showed the formation of PCO, as in human cataract surgery. In other experimental animal models, such as rodent models of extra-capsular crystalline lens extraction without IOL implantation, multiple layers of lens epithelial cells develop in the center of the posterior capsule at day 3, and the residual lens epithelial cells form a new lens only 2 weeks after surgery [14]. By contrast, in the rabbit model, PCO forms in the eye with IOL implantation at 2 to 3 weeks after the surgery [21]. Similarly, in our model a single layer of migrated cells was observed on the posterior capsule at day 5 postoperatively. Previous and present data suggest that PCO occurs more frequently and earlier in rodent eyes than in rabbit eyes. These facts led us to the idea that the light exposure experiment with the IOL-implanted mice ought to be conducted on the day following the surgery.

The Chesapeake Bay Watermen Study reported the relationship between light exposure and the incidence of macular degeneration for the first time [22]. Since then, epidemiological studies have indicated the relationship between light exposure and retinal diseases, and prospective studies have shown that light exposure is a risk factor of early AMD [8,9,23]. However, other reports have disclaimed the relationship between sun exposure and macular degeneration [24]. Hence, the link between phototoxicity and AMD is still controversial and elucidation of the risk after cataract surgery on AMD development is important. Previously, other groups have reported the retinal protective effects of yellow IOL materials placed in front of the eyes of experimental animals without crystalline lens removal [15,16]. By contrast, in the present study, we demonstrate for the first time in vivo evidence of the protective effects of yellow IOL materials on biochemical, morphological, and electrophysiological aspects of the retinal photoreceptors using a novel murine model with crystalline lens removal and IOL implantation. The current model of IOL implantation may provide information in the future on the mechanisms of photoreceptor damage using short wavelength visible light in AMD development after cataract surgery [11].

Besides the photoreceptors, RPE cells are the other potential candidates that may play a role in retinal phototoxicity, which leads to the pathogenesis of AMD [25-27]. In this study, we sought to evaluate light-induced degeneration of the RPE; however, few apoptotic cells were found in the RPE layer after light stimulation, and no difference was detected between the clear IOL- and the yellow IOL implantation group (data not shown). This result might be due to the experimental design, such as the specific strength of the light stimulation or the time window of analysis. Further investigation of the RPE cells is required to gain a better understanding of their role in phototoxicity.

In summary, we described a new animal model of IOL implantation and demonstrated the protective effects of colored-IOL against retinal phototoxicity after cataract surgery. The data showed that our murine IOL implantation model provides a realistic representation of human cataract surgery and may facilitate the elucidation of mechanisms underlying IOL-associated issues. In future, our model will allow for the study of post-surgical phototoxicity in susceptible disease groups by using various transgenic mice or animal models of retinal disease.
